# Supplementary material for: Proton Affinity in the Chemistry of Beta-Octamolybdate: HPLC-ICP-AES, NMR and Structural Studies
Source: Molecules. 2022 Nov 30;27(23):8368. doi: 10.3390/molecules27238368 (PMC9738851; doi:10.3390/molecules27238368)
Supplement: Supplementary file 1 [file molecules-27-08368-s001.zip › molecules-2031295-supplementary.pdf]

Proton Affinity in the Chemistry of Beta-Octamolybdate: HPLC-ICP-AES, NMR and Structural Studies

Victoria V. Volchek [1], Nikolay B. Kompankov [1], Maxim N. Sokolov [1] and Pavel A. Abramov [1,2,*]

Supplementary Materials

Table of contents

Table S1. SCXRD Experimental details. 2

Figure S1. Comparison of experimental and calculated powder diffraction patterns for **1**. 3

Figure S2. ^{51}V NMR spectrum of $(Bu_4N)_3Na[V_2Mo_4O_{19}]$ (DMSO-d_6, r.t.) 4

Figure S3. ^{51}V NMR spectrum of the reaction mixture containing 70 mg **Mo8** (300 μL DMSO-d_6) + 55 mg **V10** (300 μL DMSO-d_6). 5

Figure S4. ^{51}V NMR spectrum of the reaction mixture containing 70 mg **Mo8** (300 μL DMSO-d_6) + 22 mg **V10** (300 μL DMSO-d_6), after 10 min at 60 °C. 5

Table S1. SCXRD Experimental details.

	**1**
Chemical formula	$C_{53}H_{114}AgMo_8N_5O_{26}$
M_r	2112.88
Crystal system, space group	Triclinic, $P\bar{1}$
Temperature (K)	150
a, *b*, *c* (Å)	11.977 (3), 12.363 (3), 14.719 (3)
α, β, γ (°)	70.953 (7), 67.377 (6), 82.193 (7)
V (Å^3)	1901.5 (7)
Z	1
Radiation type	Mo $K\alpha$
μ (mm^{-1})	1.60
Crystal size (mm)	0.24 × 0.13 × 0.10
Diffractometer	Bruker D8 Venture diffractometer
Absorption correction	Multi-scan<br>*SADABS* 2016/2: Krause, L., Herbst-Irmer, R., Sheldrick G.M. & Stalke D., J. Appl. Cryst. 48 (2015) 3-10
T_{min}, T_{max}	0.664, 0.745
No. of measured, independent and observed [$I > 2\sigma(I)$] reflections	23898, 7266, 6252
R_{int}	0.045
θ values (°)	θ_{max} = 25.8, θ_{min} = 1.6
$(\sin \theta/\lambda)_{max}$ (Å^{-1})	0.613
Range of *h*, *k*, *l*	$-14 \le h \le 14$, $-15 \le k \le 14$, $-18 \le l \le 13$
$R[F^2 > 2\sigma(F^2)]$, $wR(F^2)$, S	0.061, 0.154, 1.31
No. of reflections, parameters, restraints	7266, 427, 25
H-atom treatment	H-atom parameters constrained
Weighting schene	$w = 1/[s^2(F_o^2) + 27.2093P]$, where $P = (F_o^2 + 2F_c^2)/3$
$\Delta\rho_{max}$, $\Delta\rho_{min}$ (e Å^{-3})	0.92, -1.40

Computer programs: *APEX3* (Bruker-AXS, 2016), *SAINT* (Bruker-AXS, 2016), *SHELXT* 2014/5 (Sheldrick, 2014), *SHELXL2017*/1 (Sheldrick, 2017).

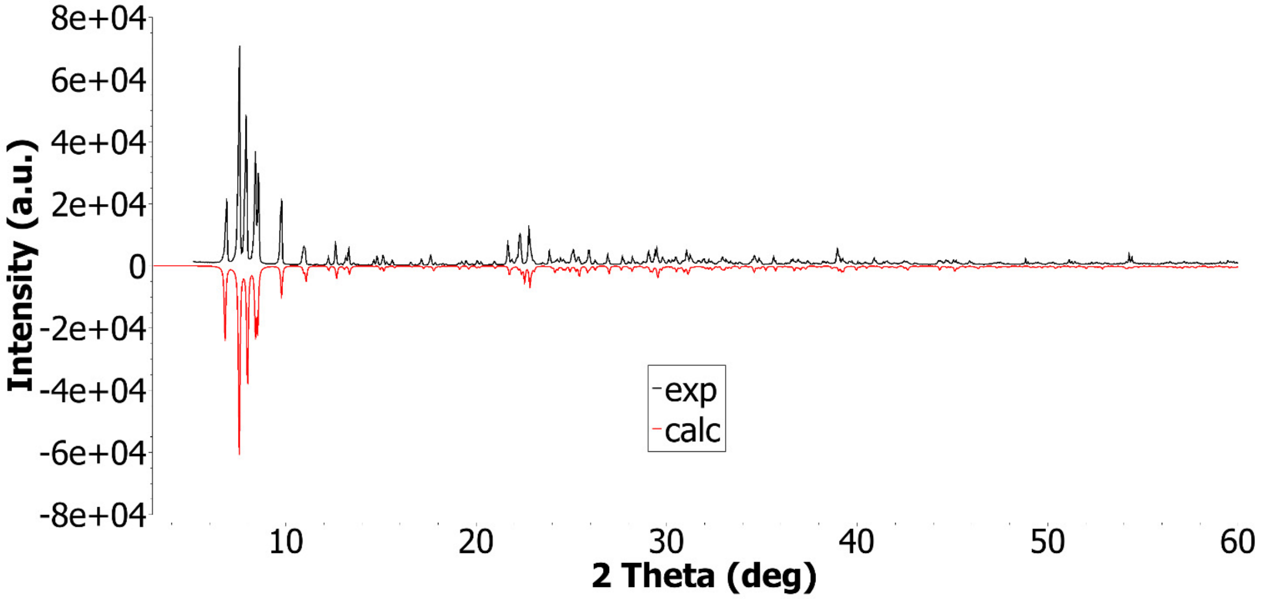

Figure S1. Comparison of experimental and calculated powder diffraction patterns for **1**.

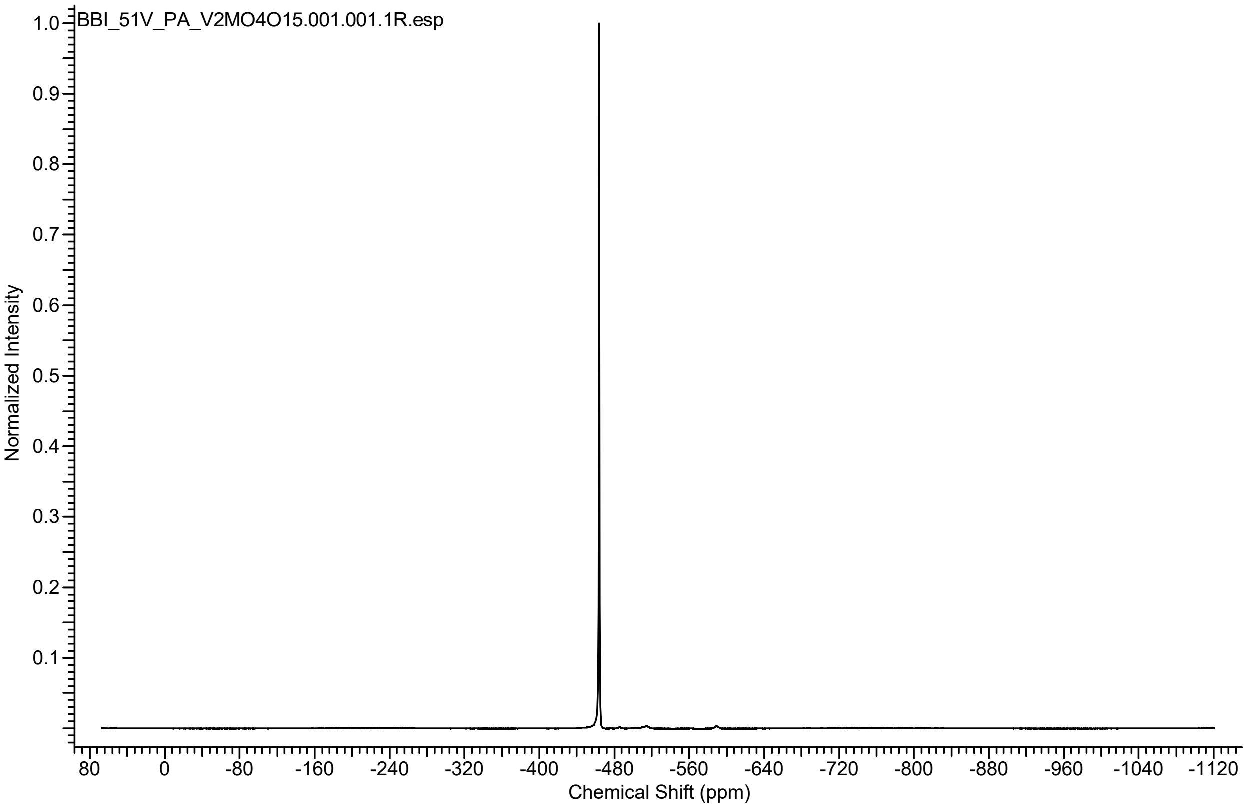

Figure S2. ^{51}V NMR spectrum of $(Bu_4N)_3Na[V_2Mo_4O_{19}]$ (DMSO-d_6, r.t.)

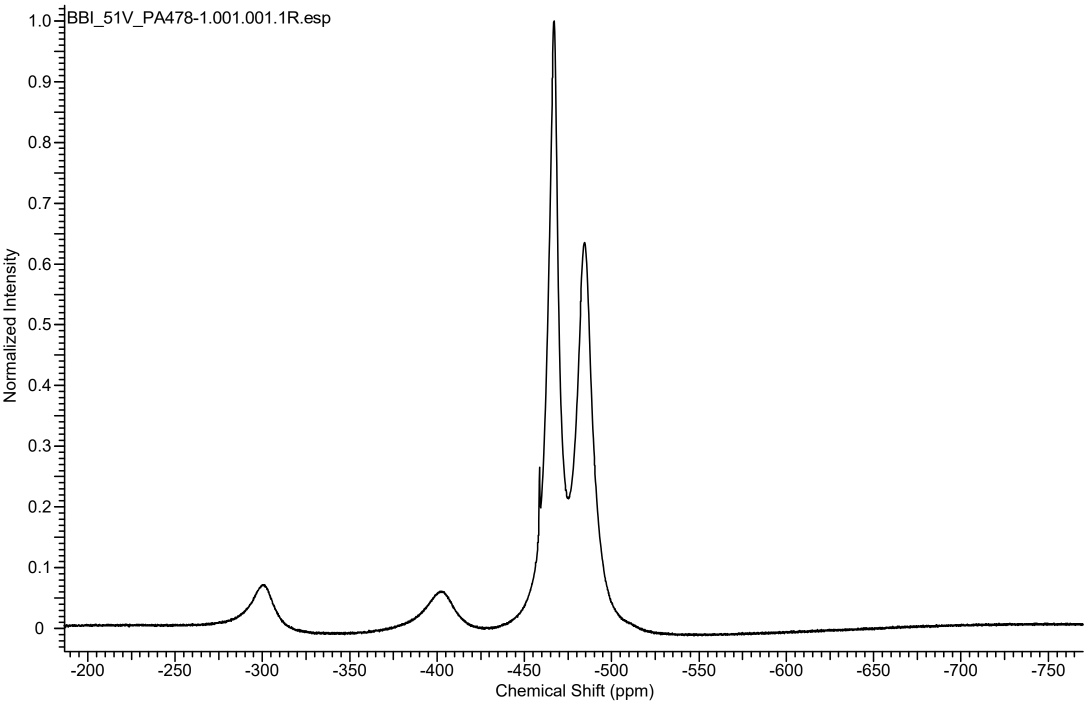

Figure S3. ^{51}V NMR spectrum of the reaction mixture containing 70 mg **Mo8** (300 µL DMSO-d_6) + 55 mg **V10** (300 µL DMSO-d_6).

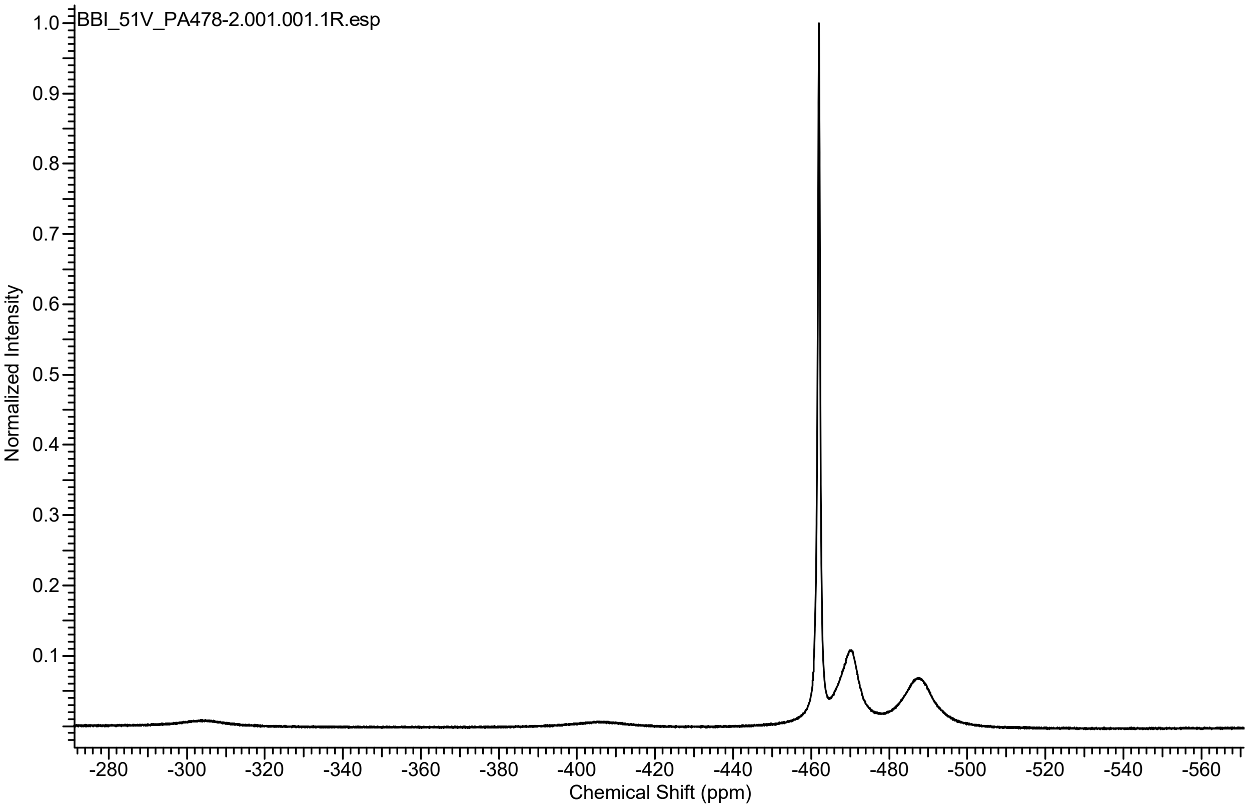

Figure S4. ^{51}V NMR spectrum of the reaction mixture containing 70 mg **Mo8** (300 µL DMSO-d_6) + 22 mg **V10** (300 µL DMSO-d_6), after 10 min at 60 °C.
